# The effect of working memory training on patient and informant reported executive function in mild cognitive impairment: an interventional study

**DOI:** 10.1186/s12883-025-04381-4

**Published:** 2025-09-30

**Authors:** Per R. Nordnes, Trine Holt Edwin, Marianne M. Flak, Gro Christine Christensen Løhaugen, Jon Skranes, Linda Chang, Haakon R. Hol, Ingun Ulstein, Susanne S. Hernes

**Affiliations:** 1 Department of Geriatrics and Internal Medicine, Sørlandet Sykehus HF, Postboks 416, Kristiansand, Lundsiden 4604 Norway; 2 Institute of Clinical Science, University of Bergen, Bergen, Norway; 3 Department of Geriatric Medicine, Oslo University Hospital, Oslo, Norway; 4 Clinic for Mental Health and Substance Abuse Treatment, Telemark Hospital Trust, Telemark County, Skien, Norway; 5 Department of Pediatrics, Sørlandet Hospital HF, Arendal, Norway; 6 Department of Clinical and Molecular Medicine, Faculty of Medicine and Health Sciences, Norwegian University of Science and Technology, Trondheim, Norway; 7 Departments of Diagnostic Radiology & Nuclear Medicine, and Neurology, University of Maryland School of Medicine, Baltimore, MD USA; 8 Department of Radiology, Oslo University Hospital HF, Oslo, Norway

**Keywords:** Mild cognitive impairment, Computerized Cognitive training, BRIEF-A, Patient-reported outcome

## Abstract

**Background:**

Self-reported outcome measures are rarely described in individuals with mild cognitive impairment (MCI). In this study, we investigated the effect of computerized working memory training on self- and relatives reported executive function measures.

**Methods:**

A total of 50 individuals with MCI were recruited from five memory clinics in Southern Norway and underwent a 5-week/20–25 sessions computerized working memory training program. Both individuals and relatives scored the “Behavior Rating Inventory of Executive Function for Adults” (BRIEF-A) before, and at least four months after finalizing training. Statistical analyses included paired t-tests and mixed ANOVA with rater type (participant/informant) and time (baseline/4-months).

**Results:**

Mixed ANOVA revealed significant (adjusted *p* ≤ 0.025) participant-informant x time interactions for Working Memory, Metacognition Index and Global Executive Composite, suggesting that the changes in ratings over time differed between participants and informants. Post hoc tests revealed that at baseline participants rated themselves as significantly more impaired (adjusted *p* ≤ 0.036) than informants on the Working Memory scale, the Metacognitive Index and the Global Executive Composite. After training, no significant differences were found between participant and informant reports. After training participants reported significant improvements on Working Memory (adjusted *p* = 0.038), Metacognition Index (adjusted *p* = 0.038), with working memory improving from above the established clinically significant impairment threshold to the normal range. The change in Global Executive Composite was not significant after correction for multiple comparisons (adjusted *p* = 0.057). No significant changes were found for relatives’ reported scores between the two time points. Excluding participants developing dementia during the study enhanced the difference in mean values before and after the intervention for these outcomes.

**Conclusion:**

Computerized working memory training significantly improved self-reported executive function in individuals with MCI. While informant reports remained stable, the prior significant discrepancy between self- and informant ratings converged after training due to this improvement. These findings suggest that training can enhance subjective cognitive experience and awareness, offering potential clinical benefits for individuals with MCI. Our study highlights self-report measures as valuable and sensitive outcomes in MCI interventions, capturing the personal experiences of cognitive change.

**Trial registration:**

ClinicalTrials.gov, NCT01991405. Registered on 18.11.2013.

**Supplementary Information:**

The online version contains supplementary material available at 10.1186/s12883-025-04381-4.

## Background

Mild cognitive impairment (MCI) represents a recognizable decline in cognitive abilities, and is currently the earliest stage for identification of cognitive deficits. Yearly, around 10% to 15% of individuals with MCI develop dementia [1]. In 2019, an estimated 57.4 million people lived with dementia globally, and this number is expected to rise to 152.8 million in 2050 [2]. Considering the high prevalence of dementia in late life, even small delays in onset could have an important impact on both the economic burden and quality of life for affected individuals and their next of kin [3].

Working memory enables the temporary storage and manipulation of information, and play an essential role in everyday cognitive function [4]. Working memory deficits are common in individuals with MCI and dementia [5, 6], and are a possible target for restorative interventions. Recent studies have shown that cognitive training appears to have an effect on varying aspects of memory in individuals with MCI, often demonstrated through objective cognitive improvements [7, 8]. On the other hand, patient reported outcome measures (PROMS) are rarely reported in cognitive training studies [9]. Changes in cognitive function might be viewed differently by the affected individual, their next of kin, and the health care personnel. The Behavior Rating Inventory of Executive Function-Adult version (BRIEF-A) [10] is an established self- and informant reported measure developed to assess different aspects of everyday executive functioning. It has been widely used in research settings, including studies on conditions such as schizophrenia [11] and traumatic brain injury [12]. The BRIEF-A has also been employed in MCI research, including a study by Rabin et al. [13] that not only identified greater self-reported executive dysfunction in MCI compared to healthy controls, but also highlighted discrepancies between self- and informant ratings with self-reported difficulties more pronounced when compared to assessments made by their informants. Moreover, impairments in the BRIEF-A Working Memory scale were found to be the most frequently reported challenge in the MCI group. This suggests that individuals with MCI have a heightened subjective awareness of their own cognitive changes, possibly as experiences of increased effort, even before these changes become observable to their informants. This finding is in notable contrast to research on other self- and informant-report questionnaires used to assess broader cognitive function in MCI, such as the Everyday Cognition (ECog) scale [14]. With the ECog, the relationship often appears to be the reverse, where informants tend to report greater impairment or more difficulties than the patients [15, 16]. To our knowledge, examination of changes in BRIEF-A after computerized working memory training in individuals with MCI has not been reported previously.

The aim of this study was to investigate changes in self-reported and informant-reported scores on BRIEF-A among individuals with MCI and mild dementia before and four months after computerized working memory training. Working memory is a foundational executive function involved in planning, problem-solving, and everyday tasks [17], and earlier studies have found positive effects of working memory training on patient-reported executive function in other clinical populations [18]. We therefore hypothesized that computerized working memory training results in improvement on BRIEF-A, particularly on the Working Memory scale, as this scale directly assesses the cognitive domain targeted by the intervention.

## Methods

The data from this study was collected in a double-blind, placebo-controlled, randomized clinical trial: Memory Aid. The study protocol is previously published [19]. This study was approved by the Norwegian Regional Committee for medical and health research ethics (2013/410/REK Sør-Øst), and informed written consent were obtained from all participants. This study was designed, conducted and reported in accordance with the Consolidated Standards of Reporting Trials (CONSORT) 2010 statement [20]. A completed CONSORT checklist, based on the CONSORT 2025 update, is provided as a supplementary file.

### Participants

The Memory Aid Study was conducted between August 2013 and December 2017; participants were recruited from memory clinics in southern Norway; Sørlandet Hospital Arendal, Diakonhjemmet Hospital Oslo, Telemark Hospital and Oslo University Hospital. At all centers, participants underwent a comprehensive diagnostic evaluation following the recommendations of the Norwegian Registry of Persons Assessed for Cognitive Symptoms (NorCog) [21], established to promote standardized diagnostic practices across memory clinics in Norwegian specialist healthcare. The evaluation involved a multidisciplinary team and included neurocognitive testing, questionnaires for risk factor identification and potential confounding psychiatric conditions, like depression, and brain magnetic resonance imaging (MRI). Following this assessment, patients were diagnosed according to the Petersen/Winblad criteria for MCI [22]: (1) Absence of dementia as defined by ICD-10 or DSM-IV criteria, (2) intact activities of daily living / minimal impairment in complex instrumental functions, (3) cognitive decline, as evidenced by self- and/or informant reports and objective cognitive decline on objective cognitive tasks, and/or longitudinal neurocognitive test performance.

### Randomization

In the original study [23], an independent researcher randomized participants to either an adaptive or non-adaptive cognitive training group using permuted block design stratified by centre. Post-study analyses revealed comparable training effects in both groups, with no significant difference between the adaptive and non-adaptive training groups on the primary outcome of non-trained working memory tasks. Additionally, there were no significant differences in secondary outcomes which included performance on other cognitive domains tested. Therefore, we combined the adaptive and the non-adaptive groups in this analysis to determine the effect of cognitive training on BRIEF-A scores.

### Cognitive training

The computerized cognitive training was done using Cogmed (Neural Assembly Int AB, Stockholm, SWE). This software consists of several game-like tasks in which the participants use visuospatial, verbal and visual working memory. In the adaptive training group, task difficulty increased progressively as participants improved, whereas the non-adaptive training group maintained a constant low level of difficulty throughout the sessions. During the training period of 5 weeks, participants were assigned to 30 – 40 min of training per day for 5 days each week. Following the Cogmed protocol, participants received a brief weekly phone call to ensure training adherence. Participants were blinded to group allocation.

### Assessment

All participants were assessed on working memory, attention, processing speed, executive function, and visual and verbal memory [23]. Also, General Ability Index (GAI) from the Wechsler Adult Intelligence Scale–IV (WAIS-IV) [24] was performed on all participants at baseline. Testing was performed by an experienced neuropsychologist at baseline (before training), one month post-training, and four months post-training. The neuropsychologist was blinded to group allocation.

The BRIEF-A is a standardized measure of executive function using informant-report and self-report forms, with a total of 75 items assessing various components of executive function. This gives nine non-overlapping, theoretically and empirically derived clinical scales combined to form the Behavioral Regulation Index (including the scales Inhibit, Shift, Emotional Control, Self-Monitor) and the Metacognition Index (including the scales Initiate, Working Memory, Plan/Organize, Task Monitor, and Organization of Materials). The Global Executive Composite is a summary score incorporating all nine clinical scales to form a summary measure. Scores are standardized using T-scores, and higher scores implies greater difficulty in daily life, with a T-score ≥ 65 considered clinically significant. The BRIEF-A also has good test–retest reliability with correlations for the clinical scales, indexes, and overall scores range from r = 0.82 to 0.94 for the self-report form and from r = 0.91 to 0.94 for the informant report form over 4 weeks [10]. Participants and informants completed the Norwegian translation of the BRIEF-A questionnaire before training, one month after intervention, and four months after intervention.

### Statistics

The BRIEF-A gives 12 different scores, but to reduce the risk of false positive results from multiple comparisons, we restricted our analysis to the Working Memory scale, the two indexes Behavioral Regulation Index and Metacognition Index, and the summary score Global Executive Composite.

To detect group differences in BRIEF-A scores between participants and informants, a mixed ANOVA with rater type (participant/informant) as a between-subjects factor and time (baseline/4-months) as a within-subjects factor was performed.

Post hoc analyses with paired t-tests were conducted to compare mean participant scores before and four months after the intervention, mean informant scores before and four months after the intervention, and to compare mean participants scores to mean informant scores before and four months after the intervention.

At the baseline assessment, nine participants were found to meet the criteria for dementia according to either ICD-10 or DMS-IV. Two additional participants developed dementia during the follow-up assessments. To investigate the effect of cognitive training in participants with stable MCI, we also conducted a subgroup analysis on the same variables excluding participants with dementia. To account for multiple comparisons, we applied the Benjamini–Hochberg false discovery rate (FDR) correction separately to five families of related tests: (1) time × rater type interactions, (2) baseline participant vs. informant comparisons, (3) post-training participant vs. informant comparisons, (4) within-participant pre-post training comparisons, and (5) within-informant pre-post training comparisons. Each family contained four comparisons corresponding to the four BRIEF-A outcome measures. Statistical significance was maintained at α = 0.05 after FDR correction.

Missing data were handled using pairwise deletion for T-tests and listwise deletion for mixed ANOVA. Four data points were identified as outliers, being outside the valid range for T-scores on the assessed BRIEF-A domains and were excluded from the analysis. Mixed ANOVA were performed on IBM SPSS (IBM Corp, version 29.0) and T-tests were performed on STATA/SE (StataCorp. LLC, version 17.0) for windows.

## Results

### Study sample

**Fig. 1:**
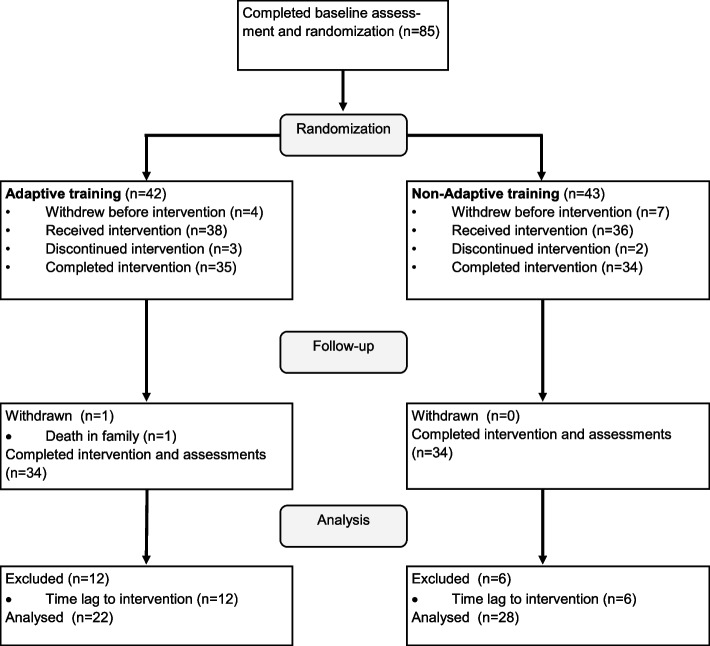
Flowchart of recruited participants and number of participants that completed intervention, follow up, and analysis

A total of 85 eligible participants were recruited between August 2013 and December 2017. All participants underwent a baseline assessment with neurocognitive testing. Eleven participants withdrew from the study prior to the training. Of the remaining 74 participants, five withdrew after they started training due to difficulty or boredom. Two participants withdrew before final assessment: one due to unrelated health causes, and one due to death in the family. Of the remaining 67 participants, 64 completed the training program and all clinical assessments. Eighteen participants were excluded from the analysis due to a delay of more than 300 days between baseline assessment and intervention, comparison between the excluded participants and the participants included in the analysis showed no significant differences at baseline (Supplementary Table s.1). For the final analysis, 50 participants were included (Fig. 1).

Table 1 shows the baseline characteristics these 50 participants. The mean age at baseline was 66 years and 32% were women and 68% men. Mean time between baseline and final assessment was 236 days (range 124 – 290).

**Table 1:** Baseline characteristics in the two randomized groups

	Total group *n* = 50	Adaptive Training *n* = 22	Non-adaptive training *n* = 28	*p*-value / χ2
Age at baseline, mean years (range, SD)	66 (43 – 88, 8.7)	65 (51 – 82, 7.9)	67 (43 – 88, 9.6)	0.49
Sex, Men/women (percent)	34 (68) / 16(32)	17 (77) /5 (23)	17 (61) / 11 (39)	0.47
Education, mean years (range, SD)	13.0 (7 −18, 2.8)	13.3 (7 – 18, 2.9)	12.9 (8 – 17, 2.8)	0.64
Socioeconomic status*, mean (SD)	3.4 (1.2)	3.5 (1.3)	3.4 (1.2)	0.71
Full scale Intelligence Quotient**, mean (SD)	97 (13.2)	97 (11.9)	97 (14.4)	0.99
Working Memory scale**, mean (SD)	92 (12.7)	92 (9.0)	93 (15.2)	0.76
Dementia at baseline (percent)	9 (18)	2 (9)	7 (25)	0.15
Incident dementia at 4 months (percent)	2 (5)	0 (0)	2 (7)	0.16

Means compared using independent t-test, proportions compared using Pearson's χ2 tests. Ordinal data compared using Mann–Whitney U test. *Socioeconomic status from Hollingshead’s index of education and occupational position, scale from 1 (low) to 5 (high) [25].**from WAIS-IV, Wechsler Adult Intelligence scale, 4th edition

*Abbreviations: SD* standard deviation

### Comparison of self-reported and informant-reported BRIEF-A scores before and after cognitive training

After correction for multiple comparisons, significant time x group interaction was observed for Working Memory (F(1, 38) = 7.220, adjusted *p* = 0.022, η2ₚ = 0.160), Metacognition Index (F(1, 38) = 8.957, adjusted *p* = 0.02, η2ₚ = 0.195) and Global Executive composite (F(1, 38) = 6.052, adjusted *p* = 0.025, η2ₚ = 0.137). No significant interaction was observed for the Behavioral Regulation Index (F(1, 38) = 1.503, *p* = 0.228, η2ₚ = 0.038). The degrees of freedom for the error term varied slightly across the outcomes due to missing data handled via listwise deletion.

When comparing participant- and informant BRIEF-A T-scores after FDR correction, we found that prior to training, participants reported significantly more difficulties than their informants in three areas: The Working Memory scale was higher in the participants with a mean of 68.3 ± 12.9 compared to the informants’ mean of 61.0 ± 10.1 (t(44) = 3,40, adjusted *p* = 0.004), the mean difference was 7.3 points (95% CI [3.0,11.6]), with a medium effect size (d = 0.51, 95% CI [0.19,0.82]).The participant group also demonstrated a higher average on the Metacognitive Index with mean T-score 59.1 ± 10.6 compared to the informants’ mean of 55.1 ± 8.6 (t(44) = 2.41, adjusted *p* = 0.027), the mean difference was 4.0 points (95% CI [0.7,7.4]), with a small effect size (d = 0.36, 95% CI [0.06,0.66]). Finally, the participants’ T-scores on the Global Executive Composite with a mean of 57.1 ± 10.5 was higher than the informants scores with a mean 53.3 ± 7.9 (t(44) = 2.46, adjusted *p* = 0.036), the mean difference was 3.8 points (95% CI [0.68,6.88]), with a small effect size (d = 0.37, 95% CI [0.06,0.67]). No significant difference was found on the Behavior Regulation Index. After training, no significant difference was found when comparing results from participants and informants (Table 2).

**Table 2:** Comparison of participant vs. informant BRIEF-A T-scores, before and after cognitive training

	Before Training			After Training			Rater type x time—*p* value (BH Adjusted)
	Participants Mean(SD)	Informants Mean(SD)	*p* (BH Adjusted)	Participants Mean(SD)	Informants Mean(SD)	*p* (BH adjusted)	
Behavior Regulation Index	53.4 (10.4)	50.7 (7.7)	.069 (0.069)	52.2 (12.3)	51.4 (8.2)	.629 (1.0)	.228 (0.228)
Working Memory	68.3 (12.9)	61.0 (10.1)	.001** (0.004)**	65.2 (14.7)	62.4 (12.6)	.230 (0.92)	.011 **(0.022)**
Metacognition Index	59.1 (10.6)	55.1 (8.6)	.020 **(0.027)**	56.3 (11.7)	56.7 (10.7)	.848 (1.0)	.005 **(0.02)**
Global Executive Composite	57.1 (10.5)	53.3 (7.9)	.018 **(0.036)**	54.9 (12.0)	54.6 (9.6)	.873 (1.0)	.019 **(0.025)**

Mean T-scores compared using paired t-tests and ANOVA with rater type (participant/informant) as a between-subjects factor and time (baseline/4-months) as within-subjects factor. Bold text signifies *p* < .05 after Benjamini–Hochberg correction for multiple comparisons within each comparison family

*Abbreviation*: *SD* standard deviation

### Comparison of BRIEF-A scores before versus after cognitive training among participants and informants

For the participants, the BRIEF-A T-score on the Working memory scale significantly decreased from a mean of 68.4 ± 12.9 at baseline to 64.7 ± 14.5 after cognitive training (t(45) = 2.61, adjusted *p* = 0.038), the mean difference was 3.7 points (95% CI [0.86,6.62]), with a small to medium effect size (d = 0.39, 95% CI [0.08,0.68]). Similarly, scores on the Metacognitive Index showed a significant reduction, with a baseline mean of 58.8 ± 10.6 compared to 56.0 ± 11.7 after intervention (t(44) = 2.44, adjusted *p* = 0.038), the mean difference was 2.8 points (95% CI [0.49,5.11]), with a small effect size (d = 0.36, 95% CI [0.06,0.66]). The improvement in Global Executive Composite from a baseline mean T-score of 56.9 ± 10.4, to a mean of 54.5 ± 12.2 after cognitive training was not significant after corrections for multiple comparisons (uncorrected *p* = 0.043, adjusted *p* = 0.057), the mean difference was 2.4 points (95% CI [0.08,4.71]), with a small effect size (d = 0.31, 95% CI [0.01,0.60]). No significant difference was found on the Behavior Regulation Index. For the informants, no significant difference was found when comparing BRIEF-A scores before and after training (Table 3).

**Table 3:** Comparison of BRIEF-A T-scores before vs. after cognitive training among participants and informants

	Participants			Informants		
	Before Training	After Training	*p* (BH Adjusted)	Before Training	After Training	*p* (BH Adjusted)
Behavior Regulation Index, mean (SD)	53.3(10.3)	51.9(12.3)	.270 (1.0)	50.6(7.8)	51.8(8.2)	.197 (0.263)
Working Memory, mean (SD)	68.4(12.9)	64.7(14.5)	.012 **(0.038)**	61.5(10.3)	62.9(12.6)	.328 (0.328)
Metacognition Index, mean (SD)	58.8(10.6)	56.0(11.7)	**.**019** (0.038)**	55.5(9.0)	57.0(10.3)	.121 (0.263)
Global Executive Composite, mean (SD)	56.9(10.4)	54.5(12.2)	.043 (0.057)	53.5(8.2)	54.9(9.4)	.137 (0.263)

Mean T-scores compared using paired t-tests, Bold text signifies *p* < .05 after Benjamini–Hochberg correction for multiple comparisons within each comparison family

*Abbreviation:* SD standard deviation

### Subgroup analyses

To assess the effect on individuals with stable MCI, the dataset was reanalyzed without the eleven participants with dementia (n = 39).

#### Comparison of participant with stable MCI versus their informant BRIEF-A scores, before and after cognitive training

The FDR corrected mixed ANOVA with rater type (participant/informant) as a between-subjects factor and time (baseline/4-months) as a within-subjects factor revealed a significant rater type × time interaction for Working Memory (F(1, 30) = 9.214, adjusted *p* = 0.01, η2ₚ = 0.235), Metacognition Index (F(1, 29) = 10.466, adjusted *p* = 0.012, η2ₚ = 0.265) and Global Executive composite (F(1, 30) = 6.566, adjusted *p* = 0.021, η2ₚ = 0.180). No significant interaction was observed for the Behavioral Regulation Index (F(1, 30) = 1.405, adjusted *p* = 0.245, η2ₚ = 0.045). The degrees of freedom for the error term varied slightly across the outcomes due to missing data handled via listwise deletion.

In the subgroup with stable MCI, before training, participants had a significantly higher BRIEF-A T-score on the Working Memory scale, the Metacognition Index, and the Global Executive Composite, compared to informants. For the Working Memory scale, before training, participants had a mean T-score of 69.1 ± 13.8, while the mean of the informants were 59.1 ± 10.0 (t(34) = 4.03, adjusted *p* = < 0.001), the mean difference was 10.0 points (95% CI [5.3,14.7]), with a medium to large effect size (d = 0.73, 95% CI [0.35,1.10]). On the Metacognition Index participants had a mean T-score of 59.6 ± 11.7, while informants had a mean of 53.5 ± 8.6 (t(34) = 3.14, adjusted *p* = 0.006), the mean difference was 6.1 points (95% CI [2.2,10.0]), with a medium effect size (d = 0.53, 95% CI [0.17,0.88]). On the Global Executive Composite participants had a mean T-score of 57.4 ± 11.5, while the informants had a mean of 52.3 ± 8.3 (t(34) = 2.90, adjusted *p* = 0.008), the mean difference was 5.1 points (95% CI [1.5,8.7]), with a medium effect size (d = 0.49, 95% CI [0.14,0.84]). No significant difference was found on the Behavior Regulation Index before training. After training no significant difference was found when comparing results from participants and informants (Table 4).

**Table 4:** BRIEF-A scores for participants with stable MCI and informants before and after training

	Before Training			After Training			Rater type x time—*p* value (BH Adjusted)
	Participants Mean(SD)	Informants Mean(SD)	p (BH Adjusted)	Participants Mean(SD)	Informants Mean(SD)	p (BH Adjusted)	
Behavior Regulation Index, mean (SD)	53.5 (11.1)	50.5 (8.3)	.063 (0.063)	52.6 (12.9)	51.3 (8.7)	.525 (0.818)	.245 (0.245)
Working Memory, mean (SD)	69.1 (13.8)	59.1 (10.0)	< .001 **(< 0.001)**	64.9 (15.2)	60.5 (12.5)	.084 (0.336)	.005** (0.01)**
Metacognition Index, mean (SD)	59.6 (11.7)	53.5 (8.6)	.003 **(0.006)**	55.6 (12.5)	55.1 (10.4)	.818 (0.818)	.003** (0.012)**
Global Executive Composite, mean (SD)	57.4 (11.5)	52.3 (8.3)	.006 **(0.008)**	54.6 (12.8)	53.6 (9.8)	.645 (0.818)	.016** (0.021)**

Mean T-scores compared using paired t-tests and ANOVA with rater type (participant/informant) as a between-subjects factor and time (baseline/4-months) as within-subjects factor. Bold text signifies *p* < .05 after Benjamini–Hochberg correction for multiple comparisons within each comparison family

*Abbreviation***:** *SD* standard deviation

#### BRIEF-A scores before versus after cognitive training among participants with stable MCI and their informants

When examining scores on the BRIEF-A before and after training for the participants with stable MCI, a significant improvement was observed in the Working Memory scale, The Metacognition Index, and the Global Executive Composite. Participants scores on the Working Memory Scale significantly decreased from a mean T-score of 69.5 ± 13.4 before training, to 64.1 ± 15.1 after training (t(36) = 3.60, adjusted *p* = 0.004), the mean difference was 5.4 points (95% CI [2.4,8.5]), with a medium effect size (d = 0.59, 95% CI [0.24,0.94]). Similarly, scores on the Metacognitive Index showed a significant reduction, with a baseline mean T-score of 59.2 ± 11.6 compared to 55.3 ± 12.4 after intervention (t(35) = 2.99, adjusted *p* = 0.01), the mean difference was 4.0 points (95% CI [1.3,6.7]), with a medium effect size (d = 0.50, 95% CI [0.15,0.82]). Finally, the Global Executive Composite also had a significant fall in the mean from 57.3 ± 11.2 at baseline, to 54.1 ± 12.9 after intervention (t(36) = 2.33, adjusted *p* = 0.035), the mean difference was 3.2 points (95% CI [0.4,5.9]), with a moderate effect size (d = 0.38, 95% CI [0.05,0.72]). No significant difference was found on the Behavior Regulation Index. For the informants, no significant difference was found when comparing BRIEF-A scores before and after training (Table 5).

**Table 5:** BRIEF-A scores for participants with stable MCI and informants before and after training

	Participants			Informants		
	Before Training	After Training	*p* (BH Adjusted)	Before Training	After Training	*p* (BH Adjusted)
Behavior Regulation Index, mean (SD)	53.6 (10.9)	52.1 (12.9)	.305 (0.305)	50.3 (8.4)	51.3 (8.9)	.286 (0.381)
Working Memory, mean (SD)	69.5 (13.4)	64.1 (15.1)	< .001** (0.004)**	59.7 (10.3)	60.8 (12.7)	.482 (0.482)
Metacognition Index, mean (SD)	59.2 (11.6)	55.3 (12.4)	.005 **(0.01)**	53.8 (9.1)	55.1 (10.1)	.276 (0.381)
Global Executive Composite, mean (SD)	57.3 (11.2)	54.1 (12.9)	.026** (0.035)**	52.4 (8.7)	53.6 (9.7)	.286 (0.381)

Mean T-scores compared using paired t-tests. Bold text signifies *p* < .05 after Benjamini–Hochberg correction for multiple comparisons within each comparison family

*Abbreviation***:** *SD* standard deviation

## Discussion

The significant group x time interactions for the Working Memory scale, Metacognition Index, and the Global Executive Composite indicated that cognitive training differently affected how participants and informants rated executive functioning over time. Post hoc analyses showed a significant difference between participants’ and informants’ ratings at baseline on the Working Memory scale, Metacognition Index, and the Global Executive Composite, with the participants reporting higher levels of impairment as compared to the informants. After training, no differences were found between these two groups, consistent with the significant rater type x time interactions observed in the mixed ANOVA. The participants reported mean scores above the established clinically significant impairment threshold (T-score ≥ 65) in the Working Memory scale at baseline and the mean fell to the normal range after training. The informants reported no changes in the Working Memory scale before and after training. Subgroup analyses of the stable MCI group further revealed a more pronounced patient-reported effect of training, as measured by BRIEF-A before and after the training intervention.

Our finding aligns with previous studies describing that individuals with MCI are more likely to report clinically significant executive difficulty compared to their informants [13, 26] on the BRIEF-A. Some studies have found that participants with MCI are relatively accurate in self-assessment of cognitive performance [27], but this insight appears to diminish over time [28]. The initial divergence identified between participants’ and relatives’ scores before training may be due to the participants’ heightened awareness of their own cognitive difficulties. Another possible explanation is that compensatory strategies can mask impairments in the earlier stages of decline, leading to more positive assessments by informants [29]. Given the complex and interconnected nature of the factors influencing self-reported executive function, it is likely that a combination of these mechanisms contributed to these group differences.

After working memory training, we found significant improvement in two of the four participant-reported outcomes. As expected, the most substantial effect was observed in the Working Memory scale, the primary focus of the training program. The significant improvement in the Metacognition Index may in part reflect the contribution of the Working Memory scale, as it constitutes parts of this composite score. This may also apply to the improvement in the Global Execute Composite, however, this finding was not significant after correction for multiple comparisons. It is possible that this is an effect of improvement in working memory, as studies suggest that working memory may also support executive control by providing a mental workspace that facilitates the optimization of goal-directed behavior [30]. Additionally, evidence indicates that memory-based interventions tend to be more effective at improving cognition than those targeting multiple domains [31]. These potential transfer effects, particularly how improvements in working memory might influence executive functions, warrant further investigation. No significant change was found in the Behavioral Regulation Index after training, suggesting that the cognitive training program does not target behavioral regulation skills.

Following the intervention, participant-reported BRIEF-A scores improved and converged with informant ratings. This convergence suggests that the intervention may have improved the participants’ executive function, reflected both in their self-perception of abilities and their capacity to accurately assess their cognitive function. This improvement in self-reported executive function parallels findings seen in working memory training studies in other clinical populations, such as individuals with human immunodeficiency virus [18]. The absence of corresponding change in informant reports, however, warrants consideration. First, functional deficits in mild cognitive impairment are, by definition, often subtle and may not have a significant impact on daily activities, and improvements in executive function experienced by participants might also be subtle, such as reduced effort or less mental fatigue, even if the observable outcome remains similar to an informant. Second, informants typically base their ratings on observable behaviors, and they may require a greater degree of overt functional improvement to perceive a significant difference. The cognitive training could therefore have produced meaningful subjective gains that may not be externally observable. It is also important to consider the possibility that reduced self-awareness of executive abilities due to disease progression could have influenced the self-reported scores. However, a previous study [32] reported stable self- and informant reported executive function over a one-year period for patients with MCI who did not progress to dementia, indicating that self-perception remains consistent over time in patients with stable MCI. Also, the subgroup analysis demonstrated a larger mean improvement in participants with stable MCI compared to the overall study population, which included 11 patients who progressed to dementia during the study, suggesting that the observed improvements were particularly pronounced in individuals who maintained their MCI diagnosis. The lack of a control group in this study also limits the attribution of these improvements to the working memory training. However, the absence of significant change in the Behavioral Regulation Index, a domain not targeted by the training, suggests that the effect is unlikely to be solely due to a placebo effect. These findings highlight the potential for greater responsiveness to cognitive interventions in individuals with stable MCI.

To our knowledge, this is the first study to report BRIEF-A as an outcome measure in individuals with cognitive decline undergoing computerized working memory training. In interventional studies targeting MCI and dementia, the majority focus on performance-based tests of cognitive function, with only a few investigating functional performance [9]. This focus on cognitive outcomes may overlook outcomes that are more meaningful to the participants and their informants [33]. Supporting this concern, Rabin et al. found that individuals with MCI reported executive function impairments on BRIEF-A despite a lack of clinically meaningful difficulties on several neuropsychological tests of executive functions [13]. Therefore, BRIEF-A may capture distinct aspects of executive function not fully assessed by traditional neuropsychological tests. Since executive function rating scales appear to reflect real-world executive functioning [34], self-reported executive function could be a valuable tool, providing a direct assessment of perceived cognitive abilities by the participant and their informants. Given that everyday function is a crucial aspect of quality of life for individuals living with MCI, incorporating the BRIEF-A into assessments could lead to a more comprehensive and nuanced understanding of their cognitive challenges.

This exploratory study has some limitations. It is important to note that while this Norwegian translation is in common use among Norwegian neuropsychologists, a formal psychometric validation with a large, representative Norwegian-speaking population has not yet been published. Studies such as Løvstad et al. [35] indicate that the U.S. normative data used in Norway should be interpreted with caution, as a group of healthy Norwegian respondents reported BRIEF-A scores ½—¾ SD below the U.S. normative mean. In the present study, our focus is the change in scores over time within the same individuals, which mitigates the impact of potential normative differences. However, the interpretation of our results related to T-score cutoffs for clinical significance, is dependent on the U.S. normative sample and should be viewed in light of this. Further, the participants were homogenous in terms of ethnicity and place of residence, which limits the generalizability of the findings to other populations. Also, the concept of MCI captures a heterogeneous group of individuals, ranging from those in the prodromal stages of dementia to those who may never develop dementia. This heterogeneity may have obscured some of the associations of interest. In addition, the study is limited by its small sample size, and due to training effect in the non-adaptive group, the study has no control group. Finally, the selection of participants might have introduced bias, as those who consented to the intervention may represent a more motivated subgroup of the MCI population.

The findings and limitations in this study highlight the need for further research on the effects of cognitive training in individuals with MCI. The ongoing Repeated Advanced Cognitive Training in Mild Cognitive Impairment (REACT MCI) study addresses some of these limitations by including an active control group, a larger number of participants, and a longer follow-up period. These improvements aim to provide more robust data, contributing to a deeper understanding and better management of MCI.

## Conclusions

This study highlights the impact of a computerized working memory training program on self-reported executive function in individuals with MCI. Our findings demonstrate that participants reported significant improvement in working memory and other executive function domains. The marked discrepancy observed at baseline, where participants perceived more executive dysfunction than their informants, resolved after training as self- and informant-reports converged. This effect was particularly pronounced in individuals with stable MCI.

These results underscore the importance of incorporating the patient’s perspective in evaluating cognitive interventions. The observed subjective improvement suggests that working memory training can enhance a person’s awareness and perceived competence in daily executive functioning, and offer clinical benefits that may not be observable to others. This underscores the potential of cognitive interventions to improve the experience and quality of life for individuals with MCI.

As the first study to report BRIEF-A as an outcome measure in cognitive training for patients with MCI, this study shows the potential of self-reported executive function as a valuable and sensitive outcome for MCI interventions. It captures crucial improvements that may not always be reflected in objective performance. Further research should investigate these benefits in larger, controlled trials to broaden our understanding.

## Supplementary Information

Supplementary Material 1.

Supplementary Material 2.

## Data Availability

Data sharing is not possible due to the incision of identifiable patient information.

## Abbreviations

BRIEF-A: Behavior Rating Inventory of Executive Function for Adults
DMS-IV: The Diagnostic and Statistical Manual of Mental Disorders, Fourth Edition
ICD-10: International Statistical Classification of Diseases and Related Health Problems 10th Revision
MCI: Mild cognitive impairment
NorCog: The Norwegian Registry of Persons Assessed for Cognitive Symptoms
PROMS: Patient reported outcome measures
SD: Standard deviation
WAIS-IV: Wechsler Adult Intelligence scale, 4th edition
