# Supplementary material for: The effect of working memory training on patient and informant reported executive function in mild cognitive impairment: an interventional study
Source: BMC Neurol. 2025 Sep 30;25:404. doi: 10.1186/s12883-025-04381-4 (PMC12487203; doi:10.1186/s12883-025-04381-4)
Supplement: Supplementary file 1 — Supplementary Material 1. [file 12883_2025_4381_MOESM1_ESM.docx]

**Table s.1** Comparison between included and excluded participants at baseline

|  | Included  n = 50 | Excluded  n = 18 | p-value / χ2 |
| --- | --- | --- | --- |
| Age at baseline, mean years (range, SD) | 66 (43 – 88, 8.7) | 67 (51 – 83, 9.5) | 0.85 |
| Sex, Men/women (percent) | 34 (68) / 16(32) | 12 (67) / 6 (33) | 0.91 |
| Education, mean years (range, SD) | 13.0 (7 -18, 2.8) | 13.4 (8 – 20, 3.3) | 0.68 |
| Socioeconomic status*, mean (SD) | 3.4 (1.2) | 3.2 (1.1) | 0.43 |
| Full scale Intelligence Quotient**, mean (SD) | 97 (13.2) | 96 (15.2) | 0.66 |
| Working Memory scale**, mean (SD) | 92 (12.7) | 90 (13.6) | 0.54 |
| Behavior Regulation Index, mean (SD) | 53.5 (10.0) | 54.7 (9.3) | 0.67 |
| Working Memory, mean (SD) | 68.5 (12.6) | 67.9 (13.0) | 0.90 |
| Metacognition Index | 58.9 (10.2) | 59.8 (13.0) | 0.77 |
| Global Executive Composite, mean (SD) | 57.0 (10.1) | 57.7 (10.9) | 0.79 |

Means compared using independent t-test, proportions compared using Pearson's χ2 tests. Ordinal data compared using Mann-Whitney U test. *Socioeconomic status from Hollingshead’s index of education and occupational position, scale from 1 (low) to 5 (high). **from WAIS-IV, Wechsler Adult Intelligence scale, 4th edition.

Abbreviations: SD; standard deviation.
